# A Sub-acute Cerebral Contusion Presenting with Medication-resistant Psychosis

**DOI:** 10.7759/cureus.2938

**Published:** 2018-07-06

**Authors:** Ashley N Rubin, Eduardo D Espiridion, Daniel H Lofgren

**Affiliations:** 1 Family Medicine, West Virginia School of Osteopathic Medicine, Lewisburg, USA; 2 Psychiatry, Frederick Memorial Hospital, Frederick, USA; 3 Surgery Student, West Virginia School of Osteopathic Medicine, Lewisburg , USA

**Keywords:** traumatic brain injury (tbi), cerebral contusion, psychosis, insomnia

## Abstract

The most common symptoms of a cerebral contusion include headache, dizziness, concentration problems, and memory loss. Insomnia is reported by more than half of the patients and can exacerbate symptoms. A 24-year-old previously healthy male presented with psychosis, acute personality changes, auditory and visual hallucinations three weeks after falling 15 feet with concurrent head trauma. A right-sided cerebral contusion with concussion was diagnosed on initial admission with increasing homicidal and suicidal ideations after 26 hours of insomnia. The patient accomplished rest after seven days of medication-resistant insomnia with the final combination of ziprasidone and lorazepam. After one night of sleep, the patient was alert and oriented with normal mood, affect, and cognition. The insomnia appeared to exacerbate this patient’s symptoms, and an atypical insomnia treatment regimen was required to induce somnolence and restore function in this patient. The combination of this abnormal patient presentation along with the unorthodox medication regimen makes this case unique compared to other traumatic brain injury symptoms and treatments.

## Introduction

Traumatic brain injury (TBI) is a disease near the forefront of American morbidity and mortality. TBIs generally result from trauma to the head due to falls, motor vehicle accidents, and strikes to the head and they disrupt the brain’s normal functioning [1]. They can lead to a multitude of pathological findings, which include skull fractures, hematomas, and contusions [2]. In a 1999 report to the Congress, the Centers for Disease Control and Prevention estimated that over 5.3 million people suffered from permanent disability related to prior TBIs [3]. They reported an incidence of 1.5 million cases during that same year [3]. Within the next 14 years, the incidence of TBIs grew to almost 2.8 million cases [1]. In addition to the effects on a patient’s quality of life post-TBI, it has been estimated that TBIs cost anywhere from $25,174 to $81,153 per patient [4].

Cerebral contusions, which can result after a TBI, originate after impact between the brain cortex and the inner surface of the skull. They are composed of focal areas of petechial hemorrhage from brain parenchyma with surrounding edema [5-6]. These contusions usually occur near the rough inner surfaces of the skull including the frontal lobe and inferior temporal lobes [7-9]. Noncontrast-enhanced computed tomography (CT) is the standard for visualizing acute contusions and shows mixed hyperdensity and hypodensity within the superficial brain. A T2-weighted magnetic resonance imaging (MRI) is more beneficial than a CT for chronic and subacute contusions, usually revealing hyperdensity within the contusion [7-8]. Symptoms of these contusions generally include aphasia, incoordination, focal weakness, seizures, cognition, and memory problems [10-12]. When compared with a diffuse axonal injury, cortical contusions were more hemorrhagic and less associated with impaired consciousness [6].

Included in this case report is a young, previously healthy male with no significant psychiatric or past medical history who presented with hallucinations, behavioral changes, and a headache three weeks postcontusion. His presenting symptoms of auditory and visual hallucinations with suicidal ideations are unlike previously reported cases of cerebral contusion [12-13]. In addition to this rare presentation, the patient suffered from seven days of insomnia which most likely exacerbated his psychosis. In addition to his abnormal symptoms, his psychosis and insomnia were resistant to basic pharmacological treatment protocols. 

## Case presentation

A previously healthy 24-year-old male presented to the emergency department with a head injury after falling 15 feet, and he was admitted with a diagnosis of a TBI to his right lateral frontal lobe. He was monitored for four days in the neurology intensive care unit and then discharged after improvement in symptoms. Three weeks from the initial injury the patient was brought into the emergency department by his family with symptoms like insomnia, atypical aggression, psychosis, and impulsive behavior. Upon arrival in the emergency department, the patient admitted to new onset suicidal and homicidal ideations with a plan to shoot himself and the (illusory) “friend who murdered his family and robbed his home” with a loaded gun in his possession. He admitted that two days prior to re-admission, he had become frustrated upon return to work, had not slept for 26 hours, and began damaging items around his house. He admitted to increased aggression, hallucinations, and paranoid ideations.

The patient, accompanied by his mother, denied any previous family or personal psychiatric history. His mother stated his personality had become increasingly impulsive and aggressive since his previous discharge from the hospital. The patient complained of worsening auditory and visual hallucinations, insomnia, headache, and visual floaters. The patient denied nausea, emesis, weakness, gait difficulty, and focal motor defects.

The patient had an unremarkable past medical history. Hypertension was diagnosed during his initial admission and he was started on lisinopril for management. Social history included intermittent alcohol and occasional marijuana use. He denied any tobacco or other illicit drug use. He lived with his parents, was in a monogamous relationship with his girlfriend, and worked in the construction field.

Upon this admission three weeks postinjury, the patient presented with a blood pressure of 152/92 mmHg and a heart rate of 105 beats/minute. He was alert, oriented, anxious, and agitated. His five-digit forward recall was 4/5, and he was able to spell the word “WORLD” backwards and forward. The patient spoke with a normal rate and volume and without aphasia. Speech was coherent and goal-directed. Mood was anxious and affect-constricted. He had paranoid ideations with auditory and visual hallucinations. His heart had a regular rhythm without clicks or murmurs. Lungs were clear to auscultation bilaterally, and he had a soft and nontender abdomen with normoactive bowel sounds. Neurological examination revealed cranial nerves II-XII were intact bilaterally. Patient had 5/5 strength bilaterally without pronator drift. No pathological reflexes were noted.

**Figure 1:**
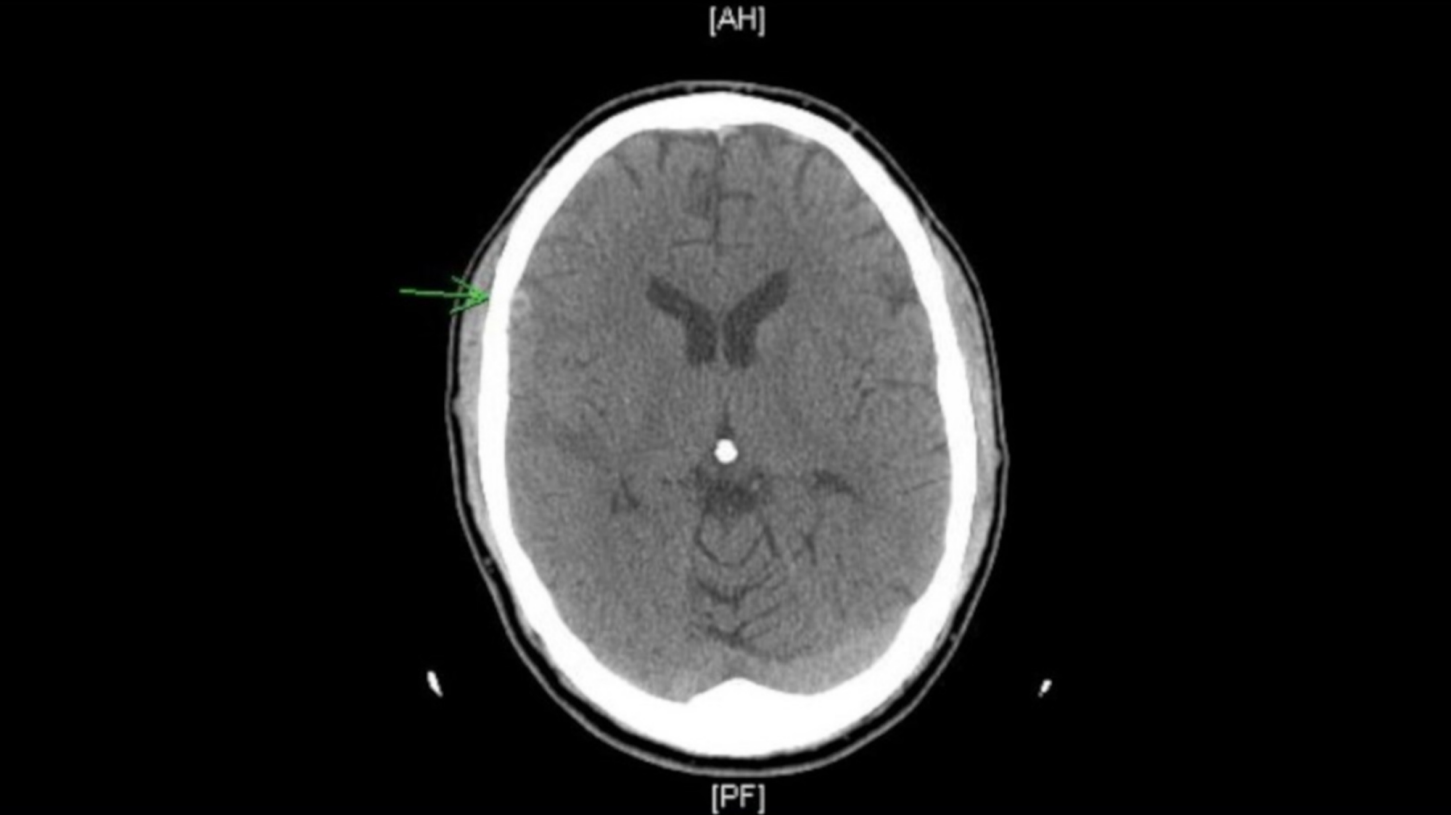
Noncontrast computed tomography (CT) of the head showing increasing cerebral edema on the right parietal lobe with no increasing mass effect or midline shift.

Upon chart review, a two-week routine follow-up CT after his initial injury revealed increasing cerebral edema on the right parietal lobe with no increasing mass effect or midline shift (Figure 1). It also noted a nondisplaced fracture of the right temporoparietal region that was not commented on prior imaging.

**Figure 2:**
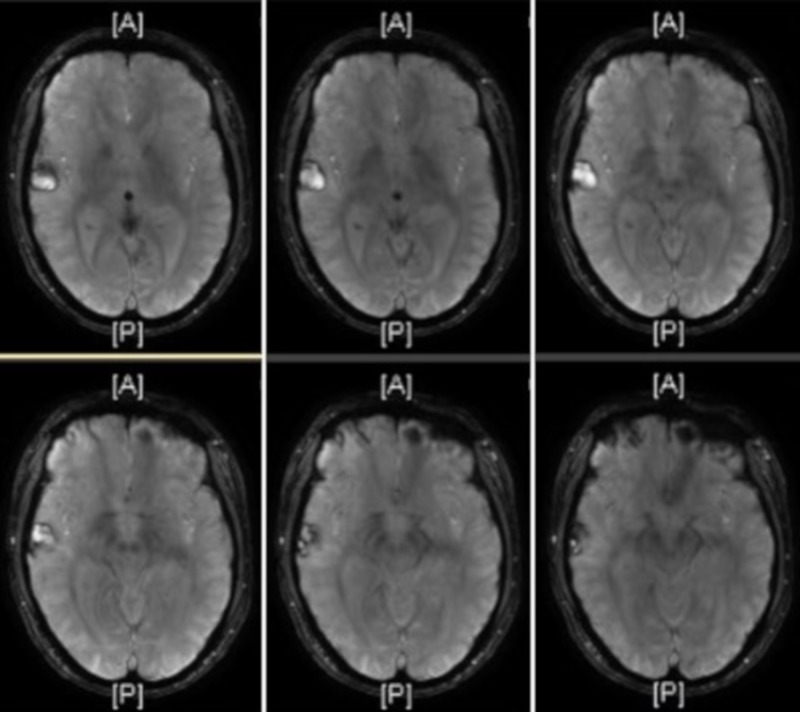
T1-weighted magnetic resonance imaging (MRI) showed several acute and subacute cerebral contusions within the right frontotemporal region with progression of the temporal lobe contusion.

**Figure 3:**
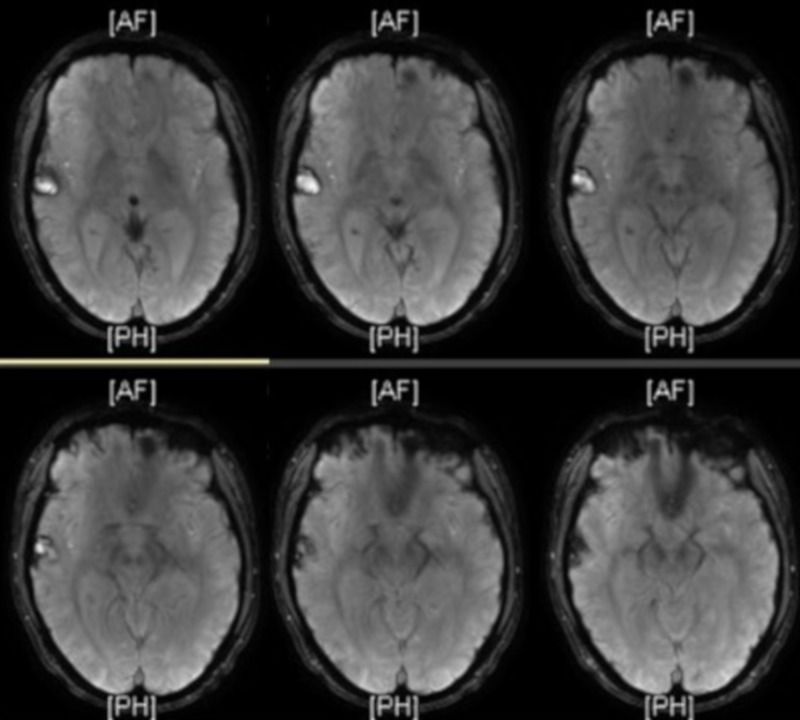
T1-weighted brain MRI revealed a persistent dominant focus in the right temporal region that was well demarcated and stable in size. It showed resolving white matter edema and improving post-traumatic foci of altered signal intensity when compared with prior imaging.

On day one of this admission, three weeks postinjury, the patient’s electroencephalography was unremarkable, and an MRI showed several acute/subacute cerebral contusions within the right frontotemporal region; with progression of the temporal lobe contusion (Figure 2). It showed mild associated perifocal edema without significant mass effect or midline shift. Four days later, a second MRI of the brain showed no significant changes. A third brain MRI was taken after the patient accomplished a full night’s rest and psychotic symptoms had resolved (Figure 3). This MRI showed a persistent dominant focus in the right temporal region that was well demarcated and stable in size. It showed resolving white matter edema and improving post-traumatic foci of altered signal intensity when compared with prior imaging.

A multi-disciplinary team was consulted which included a hospitalist, neurologist, neurosurgeon, psychiatrist, occupational therapist, physical therapist, and speech therapist. Upon this admission, the patient suffered from fluctuating auditory and visual hallucinations, anxiety, headaches, and agitation. He was started on dexamethasone, valproic acid and lorazepam, which did not alleviate his psychosis. Throughout the hospital admission, the patient reported some insight and memory to these symptoms; yet, he reported being unable to control them. Although he could recall all the events, at times he was unable to differentiate between reality and the hallucinations. Throughout the next week, a new medication was incorporated into his regimen daily to help induce sleep. These medications included high-dose trials of zolpidem, hydroxyzine, quetiapine, and olanzapine. The patient was finally able to accomplish rest with the combination of 20 mg intramuscular (IM) ziprasidone and 2 mg IM lorazepam on the seventh evening. After one night of sleep the patient was alert, oriented, cooperative with normal mood and affect. The following morning postsleep, his hallucinations and psychosis ceased completely. A few days later he was discharged to his home under family supervision with scheduled outpatient TBI rehabilitation to follow.

## Discussion

The rate of TBI-related injuries and TBI-related emergency department visits continue to rise every year [1, 5]. These injuries cause a significant financial burden and have an impact on the patient’s quality of life [4,14]. The TBI patients usually struggle with continued headaches, memory and concentration deficits. In addition, sleep-wake disturbances are reported by more than half of the patients with a TBI, but usually improve over time [14-17]. The duration of these disturbances is variable depending on the severity of injury, age, prior medical history, length of hospital stay, etc. [14-16].

Standard treatment for insomnia is the same whether or not a patient has a history of TBI. Approved medications and classes include melatonin agonists, doxepin, benzodiazepines, nonbenzodiazepine hypnotics, and suvorexant [17-19]. Not even high doses of zolpidem, hydroxyzine, quetiapine, and olanzapine helped this patient’s insomnia or concomitant psychosis. In this case, who was a drug-resistant patient, only ziprasidone with lorazepam helped induce sleep—resolving both his insomnia and psychosis. After one full night of sleep, his psychosis subsided, and he returned to baseline. He was a pleasant, calm patient with no paranoia or hallucinations after a full night’s rest.

There is no conclusive evidence to determine if the psychosis was originally caused by the head trauma or insomnia. The waxing and waning of aggressive, psychotic symptoms resolved after the insomnia resolved. The mechanism of insomnia in postcontusion and TBI patients is not well understood and more research is needed to further understand their relationship [14-15, 20].

## Conclusions

This TBI case is unique because of this patient’s abrupt change in clinical progression and medication-resistant insomnia with psychosis. He was improving daily for three weeks post-TBI and then started showing an acute, rapid decline with insomnia without definitive cause. Within two days this patient went from being healthy and happy at baseline to acting uncharacteristically aggressive, suffering from auditory and visual hallucinations, and having suicidal and homicidal ideations. Concurrent to his behavioral changes he also suffered from insomnia. A variety of factors may have contributed to his psychosis, but insomnia seemed to exacerbate his symptoms considering they increased with lack of sleep and completely ceased after accomplishing a full night’s rest. It is difficult to determine the etiology of the psychosis and how much impact his insomnia versus the TBI played in the behavioral changes. More research regarding the relationship between the commonly compromised sleep-wake cycle of patients who suffer from a TBI is needed. In conclusion, general insomnia treatment guidelines can be followed in TBI patients suffering from insomnia. Yet, in a case of medication-resistant insomnia and psychosis, a more diverse medication regimen is needed as exhibited in this study.
